# Supplementary material for: Complementary peptides represent a credible alternative to agrochemicals by activating translation of targeted proteins
Source: Nat Commun. 2023 Jan 17;14:254. doi: 10.1038/s41467-023-35951-0 (PMC9845214; doi:10.1038/s41467-023-35951-0)
Supplement: Supplementary file 1 — Supplementary Information [file 41467_2023_35951_MOESM1_ESM.docx]

Complementary peptides represent a credible alternative to agrochemicals by activating translation of targeted proteins

Ormancey and Guillotin *et al.*

**atg**gaagacgccaaaaacataaagaaaggcccggcgccattctatccgctggaagatggaaccgctggagagcaactgcataaggctatgaagagatacgccctggttc

M  E  D  A  K  N  I  K  K  G  P  A  P  F  Y  P  L  E  D  G  T  A  G  E  Q  L  H  K  A  M  K  R  Y  A  L  V

W  K  T  P  K  T  -  R  K  A  R  R  H  S  I  R  W1 K  M  E  P  L  E  S  N  C  I  R  L  -  R  D  T  P  W  F

G  R  R  Q  K  H  K  E  R  P  G  A  I1 L  S  A  G  R  W  N  R  W  R  A  T  A  -  G  Y  E  E  I  R  P  G  S

ctggaacaattgcttttacagatgcacatatcgaggtggacatcacttacgctgagtacttcgaaatgtccgttcggttggcagaagctatgaaacgatatgggctgaa

P G  T  I  A  F  T  D  A  H  I  E  V  D  I  T  Y  A  E  Y  F E  M  S  V  R  L  A  E  A  M  K  R  Y  G  L  N

L E Q L L L Q M H I S R W T S L T L S T S K C P F G W Q K L - N D M G -

W  N  N  C  F  Y  R  C  T  Y  R  G  G  H  H  L  R  -  V  L  R  N  V  R  S  V  G  R  S  Y  E  T  I  W  A  E

tacaaatcacagaatcgtcgtatgcagtgaaaactctcttcaattctttatgccggtgttgggcgcgttatttatcggagttgcagttgcgcccgcgaacgacatttat

  T  N  H  R  I  V  V  C  S  E  N  S  L  Q  F  F  M  P  V  L  G  A  L  F  I  G  V  A  V  A  P  A  N  D  I  Y

I  Q  I  T  E  S  S  Y  A  V  K  T  L  F  N  S  L  C  R  C  W  A  R  Y  L  S  E  L  Q  L  R  P  R  T  T  F I

 Y  K  S  Q  N  R  R  M  Q  -  K  L  S  S  I  L  Y  A  G  V  G  R  V  I  Y  R  S  C  S  C  A  R  E  R  H  L

aatgaacgtgaattgctcaacagtatgggcatttcgcagcctaccgtggtgttcgtttccaaaaaggggttgcaaaaaattttgaacgtgcaaaaaaagctcccaatca

N  E  R  E  L  L  N  S  M  G  I  S  Q  P  T  V  V  F  V  S  K  K  G  L  Q  K  I  L  N  V  Q  K  K  L  P  I

  M  N  V  N  C  S  T  V  W  A  F  R  S  L  P  W  C  S  F  P  K  R  G  C  K  K  F  T  C  K  K  S  S  Q  S

-  -  T  I  A  Q  Q  Y  G  H  F  A  A4 Y  R  G  V  R  F  Q  K  G  V  A  K  N  F  E  R  A  K  K  A  P  N  H

tccaaaaaattattatcatggattctaaaacggattaccagggatttcagtcgatgtacacgttcgtcacatctcatctacctcccggttttaatgaatacgattttgt

I  Q  K  I  I  I  M  D  S  K  T  D  Y  Q  G  F  Q  S  M  Y  T  F  V  T  S  H  L  P  P  G  F  N  E  Y  D  F  V

S  K  K  L  L  S  W  I  L  K  R  I  T  R  D  F  S  R  C  T  R  S  S  H  L  I  Y  L  P  V  L  M  N  T  I  L

P  K  N  Y  Y  H  G  F  -  N  G  L  P  G  I  S  V  D  V  H  V  R  H  I  S  S  T  S  R  F  -  -  I  R  F  C

gccagagtccttcgatagggacaagacaattgcactgatcatgaactcctctggatctactggtctgcctaaaggtgtcgctctgcctcatagaactgcctgcgtgaga

  P  E  S  F  D  R  D  K  T  I  A  L  I  M  N  S  S  G  S  T  G  L  P  K  G  V  A  L  P  H  R  T  A  C  V  R

C  Q  S  P  S  I4  G  T  R  Q  L  H  -  S  -  T  P  L  D  L  L  V  C  L  K  V  S  L  C  L  I  E  L  P  A  -  D

A  R  V  L  R  -  G  Q  D  N  C  T  D  H  E  L  L  W  I  Y  W  S  A  -  R  C  R  S  A  S  -  N  C  L  R  E

ttctcgcatgccagagatcctatttttggcaatcaaatcattccggatactgcgattttaagtgttgttccattccatcacggttttggaatgtttactacactcggat

 F  S  H  A  R  D  P  I  F  G  N  Q  I  I  P  D  T  A  I  L  S  V  V  P  F  H  H  G  F  G  M  F  T  T  L  G

  S  R  M  P  E  I  L  F  L  A  I  K  S  F  R  I  L  R  F  -  V  L  F  H  S  I  T  V  L  E  C  L  L  H  S  D

I L  A  C  Q  R  S  Y  F  W  Q  S  N  H  S  G  Y  C  D  F  K  C  C  S  I  P  S  R  F  W  N  V  Y  Y  T  R  I

atttgatatgtggatttcgagtcgtcttaatgtatagatttgaagaagagctgtttctgaggagccttcaggattacaagattcaaagtgcgctgctggtgccaaccct

Y  L I C  G  F  R  V  V  L  M  Y  R  F  E  E  E  L  F  L  R  S  L  Q  D  Y  K  I  Q  S  A  L  L  V  P  T  L

I - Y  V  D  F  E  S  S  -  C  I  D  L  K  K  S  C  F  -  G  A  F  R  I  T  R  F  K  V  R  C  W  C  Q  P

F D  M  W  I  S  S  R  L  N  V  -  I  -  R  R  A  V  S  E  E  P  S  G  L  Q  D  S  K  C  A  A  G  A  N  P

attctccttcttcgccaaaagcactctgattgacaaatacgatttatctaatttacacgaaattgcttctggtggcgctcccctctctaaggaagtcggggaagcggtt

F S F  F  A  K  S  T  L  I  D  K  Y  D  L  S  N  L  H  E2 I  A  S  G  G  A  P  L  S  K  E  V  G  E  A  V

Y S P  S  S  P  K  A  L  -  L  T  N  T  I  Y  L  I  Y  T  K  L  L  L  V  A  L  P  S  L  R  K  S  G  K  R  L

I L L  L  R  Q  K  H  S  D  -  Q  I  R  F  I  -  F  T  R  N  C  F  W  W  R  S  P  L  -  G  S  R  G  S  G

gccaagaggttccatctgccaggtatcaggcaaggatatgggctcactgagactacatcagctattctgattacacccgagggggatgataaaccgggcgcggtcggta

A K R F  H  L  P  G  I  R  Q  G  Y  G  L  T  E  T  T  S  A  I  L  I  T  P  E  G  D  D  K  P  G  A  V  G

P R G2 S  I  C  Q  V  S  G  K  D  M  G  S  L  R  L  H  Q  L  F  -  L  H  P  R  G  M  I  N  R  A  R  S  V

C Q E V  P  S  A  R  Y  Q  A  R2 I  W  A  H  -  D  Y  I  S  Y  S  D  Y  T  R  G  G  -  -  T  G  R  G  R  -

aagttgttccattttttgaagcgaaggttgtggatctggataccgggaaaacgctgggcgttaatcaaagaggcgaactgtgtgtgagaggtcctatgattatgtccgg

K V V P F  F  E  A  K  V  V  D  L  D  T  G  K  T  L  G  V  N  Q  R  G  E  L  C  V  R  G  P  M  I  M  S  G

K L F H F  L  K  R  R  L  W  I  W  I  P  G  K  R  W  A  L  I  K  E  A  N  C  V  -  E  V  L  -  L  C  P

S C S I F  -  S  E  G  C  G  S  G  Y  R  E  N  A  G  R  -  S  K  R  R  T  V  C  E  R  S  Y  D  Y  V  R

ttatgtaaacaatccggaagcgaccaacgccttgattgacaaggatggatggctacattctggagacatagcttactgggacgaagacgaacacttcttcatcgttgac

Y V N N  P  E  A  T  N  A  L  I  D  K  D  G  W  L  H  S  G  D  I  A  Y  W  D  E  D  E  H  F  F  I  V  D

V M - T I  R  K  R  P  T  P  -  L  T  R  M  D  G  Y  I  L  E  T  -  L  T  G  T  K  T  N  T  S  S  S  L  T

L C K Q S  G  S  D  Q  R  L  D  -  Q  G  W  M  A  T  F  W  R  H  S  L  L  G  R  R  R  T  L  L  H  R  -

cgcctgaagtctctgattaagtacaaaggctatcaggtggctcccgctgaattggaatccatcttgctccaacaccccaacatcttcgacgcaggtgtcgcaggtcttc

R L K S L  I  K  Y  K  G  Y  Q  V  A  P  A  E  L  E  S  I  L  L  Q  H  P  N  I  F  D  A  G  V  A  G  L

A - S L -  L  S  T  K  A  I  R  W  L  P  L  N  W  N  P  S  C  S  N  T  P  T  S  S  T  Q  V  S  Q  V  F

P P E V S  D  -  V  Q  R  L  S  G  G  S  R  -  I  G  I  H  L  A  P  T  P  Q  H  L  R  R  R  C  R  R  S  S

ccgacgatgacgccggtgaacttcccgccgccgttgttgttttggagcacggaaagacgatgacggaaaaagagatcgtggattacgtcgccagtcaagtaacaaccgc

P D D D A G E L  P  A  A  V  V  V  L  E  H  G  K  T  M  T  E  K  E3 I  V  D  Y  V  A  S  Q  V  T  T  A

 P  T  M  T  P  V  N  F  P  P  P  L  L  F  W  S  T  E  R  R  -  R  K  K  R  S  W  I  T  S  P  V  K  -  Q  P

 R  R  -  R  R  -  T  S  R  R  R  C  C  F  G  A  R  K  D  D  D  G  K  R  D  R  G  L  R  R  Q  S  S  N  N  R

gaaaaagttgcgcggaggagttgtgtttgtggacgaagtaccgaaaggtcttaccggaaaactcgacgcaagaaaaatcagagagatcctcataaaggccaagaagggc

K K L R G  G  V  V  F  V  D  E  V  P  K  G  L  T  G  K  L  D  A  R  K  I  R  E  I  L  I  K  A  K  K  G

R K S C A  E  E  L  C  L  W  T  K  Y  R  K  V  L  P  E  N  S  T  Q  E  K  S  E  R  S  S  -  R  P  R  R  A

E K V A R  R  S  C  V  C  G  R  S  T  E  R  S  Y  R  K T  R  R  K  K  N  Q  R3 D  P  H  K  G  Q  E  G

ggaaagatcgccgtg**taa**

G K I A V -

E R S P3 C

R K D R R V

**Supplementary Fig. 1:** Nucleotidic **l**uciferase sequence (top line) with translation in the three different frames. LUC protein is underlined and corresponds to frame 1. Peptides are in color: cPEPluc in green, and different cPEP in orange, with frame and number refereed to Fig 1d. cPEPluc with increased size are green darker from the 5aa to the 60aa. Sequences of cPEPs are retrieved in Supplementary Data 1.


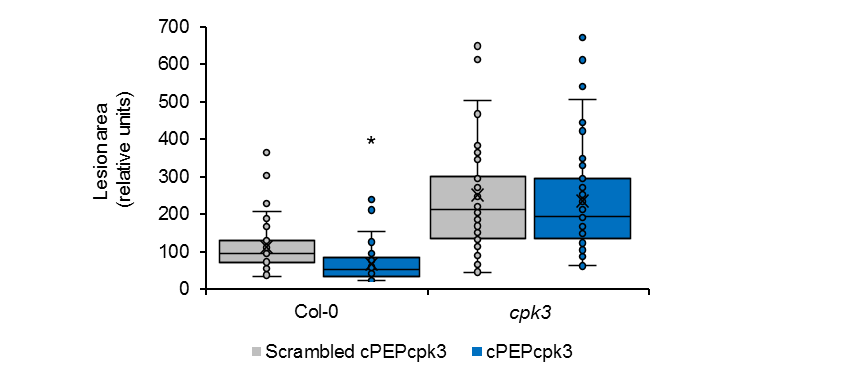


**Supplementary Figure 2**: Relative lesion area of *A. thaliana* plants infected by *B. cinerea* and treated with the indicated peptide, Col-0 and *cpk3* mutant lines. Source data are provided as source data files. For box-plots, the cross represents the mean, the line show the median value, and the upper part and lower part of box represent 1^st^ and 3^rd^ quartile. The error bars represent the minimal and maximal value. Asterisks indicate a significant difference between the test condition and the control according to the Student t-test (n = 40; p < 0.05).


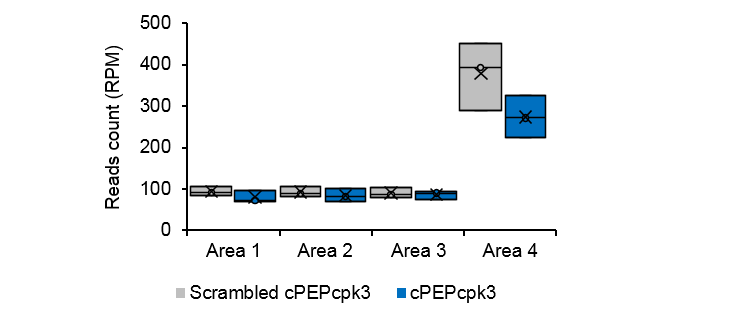

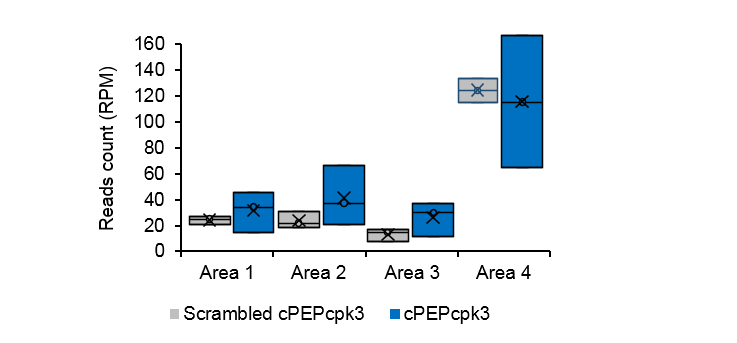


**Supplementary Figure 3**: Peptide treatment induces overaccumulation of ribosomes around CPK3 mRNA Start codon. (**a**) Metagene analysis displaying the abundance of 5’P reads in scramble and cPEP treated samples on four distinct areas. Each area is represented by a dashed line. Blue line: cPEPcpk3-HA treated samples. Orange line: scrambled cPEPcpk3-HA treated samples. (**b**) cPEPcpk3-HA treatment induces no overaccumulation of ribosomes around CPK6, CPK32 and CPK9 (close homologs of CPK3) mRNA Start codon. Quantification of 5’P reads accumulation in scramble and cPEP treated samples on four distinct areas along transcripts. Area 1 : -60 nt to –15 nt, Area 2 : -14 nt to 31 nt, Area 3 : 32 nt to 77 nt from the start codon, Area 4 : -62 nt to -17 nt from the stop codon. Error bars represent SEMs, (**c**, n = 3)

**a**

**b**

*CPK6*

*CPK32*

*CPK9*


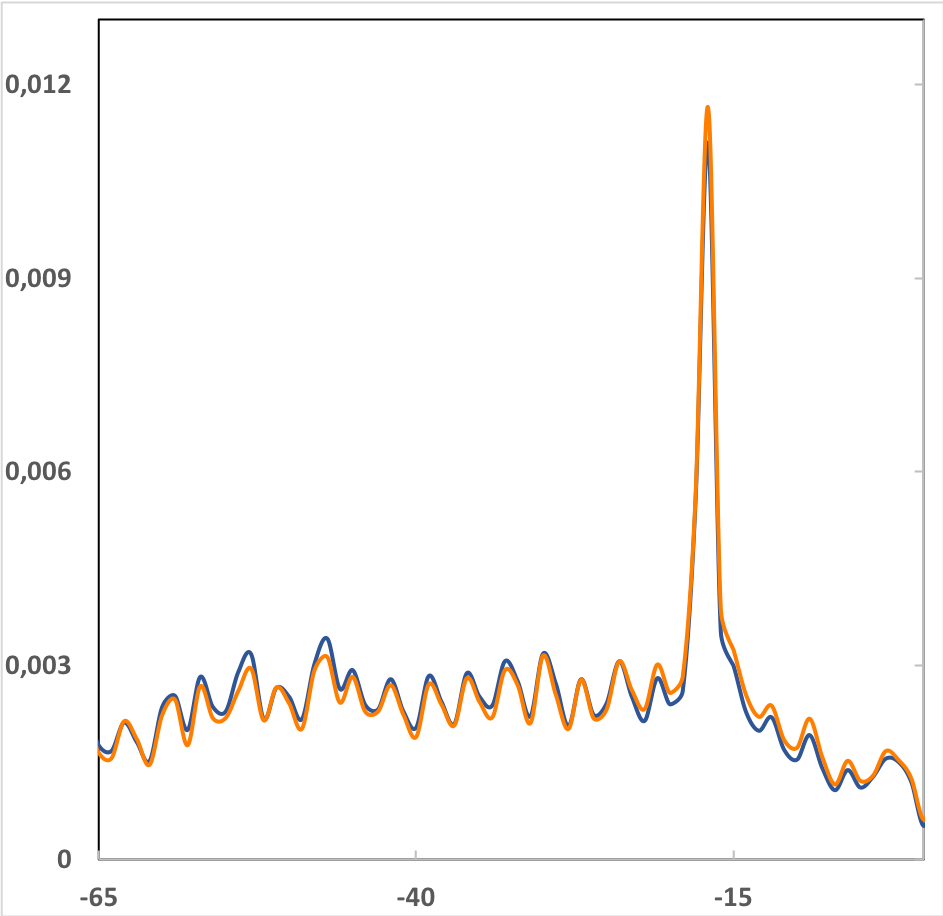


**Area 4**

-62 nt to -17 nt

**Area 1**

-60 nt to -15 nt

**Area 2**

- 14 nt to 31 nt

**ORF**

**5’UTR**

3’UTR

**AUG**

**STOP**

**Area 3**

32 nt to 77 nt


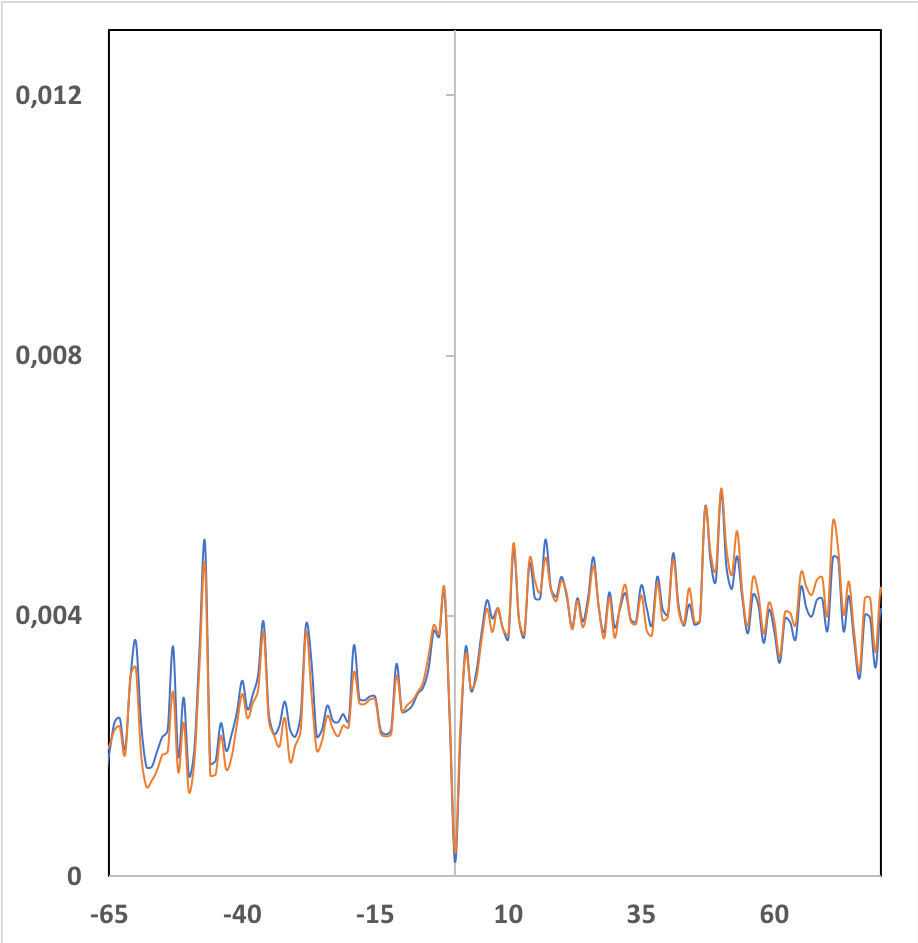


0.012

0.009

0.006

0.003

0.012

0.009

0.006

0.003

-15

-40

-65

-15

-40

-65

60

35

10

**Reads count (RPM)**

**Reads count (RPM)**


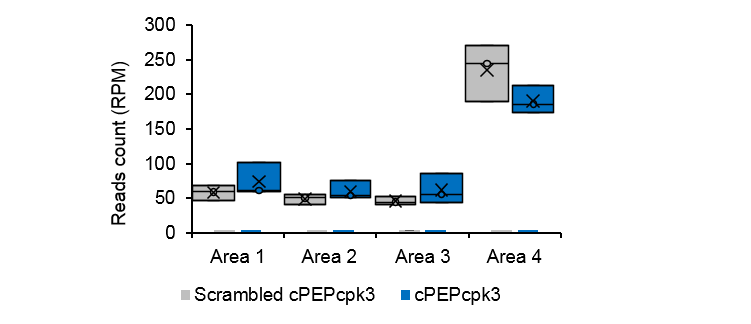


**Supplementary Figure 4**: Identification of protein partners of cPEPs by MS. (**a**) Expression of CPK3 after treatment of *A. thaliana* plants with 100 µM of cPEPcpk3-HA or Scrambled cPEPcpk3-HA, as revealed by anti-CPK3 western blot. Ponceau staining reports equal protein loading. Results are representative of three independent experiments. (**b**) Volcano plot of MS analysis.


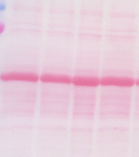

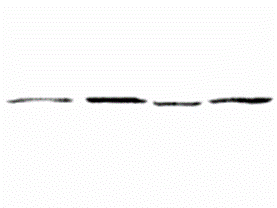


Scrambled cPEPcpk3-HA

cPEPcpk3-HA

CPK3

Rubisco


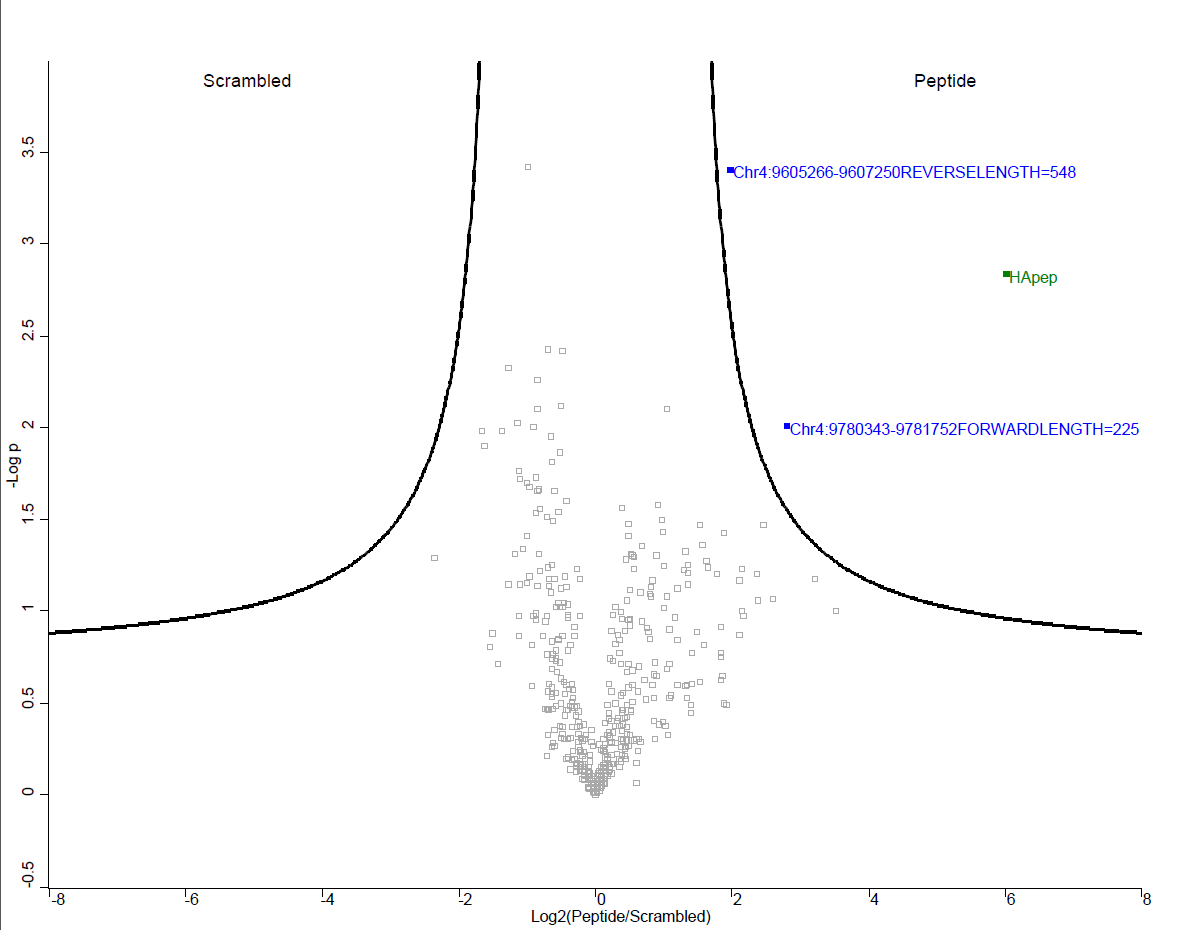


cPEPcpk3-HA

**a**

**b**

Scrambled cPEPcpk3-HA

cPEPcpk3-HA

At4g17090 (BAM3)

At4g17560 (RPL19)

| Supplementary Table 1: FRET-FLIM analysis of interaction between cPEPnsp1 and RNA |  |  |
| --- | --- | --- |

| **Donor** | **Acceptor (Sytox Orange)** | | **t ^(a)^** | | **sem ^(b)^** | | **N ^(c)^** | | **E ^(d)^** | | **p-value^(e)^** | |  |  |
| --- | --- | --- | --- | --- | --- | --- | --- | --- | --- | --- | --- | --- | --- | --- |
| **cPEPnsp1-FAM** | - | | 2.56 | | 0.028 | | 60 | | - | | - | |  |  |
| **NSP1** | + | | 2.37 | | 0.031 | | 62 | | 7.42 | | 1.615E^-05^ | |  |  |
| **cPEPnsp1-FAM** | - | | 2.78 | | 0.032 | | 39 | | - | | - | |  |  |
| **NSP1 ΔcPEP** | + | | 2.8 | | 0.037 | | 48 | | -0.719 | | 0.7545 | |  |  |
|  |  | |  | |  | |  | |  | |  | |  |  |
| Legend: | (a) mean lifetime in nanoseconds. | | | | | |  | |  | |  | |  |  |
|  | For each cell, average fluorescence decay profiles were plotted and lifetimes were estimated by fitting data | | | | | | | | | | | | | |
|  | with exponential function using a non-linear squares | | | | | | | |  | |  | |  |  |
|  | (b) standard error of the mean. | | | | | |  | |  | |  | |  |  |
|  | (c) N: total number of measured cells. | | | | | |  | |  | |  | |  |  |
|  | (d) Percentage of FRET efficiency : E=1-(tDA/tD). | | | | | | | |  | |  | |  |  |
|  | (e) p-value (Student’s t test) of the difference between the donor lifetimes in the presence or absence of acceptor. | | | | | | | | | | | | | |
| Supplementary Table 2 | | |  | |  | |  | |  | |  | |  |  |

| Protein | plant species | induction | SEM |  |  |  |
| --- | --- | --- | --- | --- | --- | --- |
| SKL | *M. truncatula* | 1,59* | 0,1 |  |  |  |
| BAK1 | *A. thaliana* | 2,65* | 0,29 |  |  |  |
| BRI1 | *A. thaliana* | 3,16* | 0,52 |  |  |  |
| CPK3 | *A. thaliana* | 5,36* | 0,46 |  |  |  |
| DCL1 | *A. thaliana* | 2,96* | 1,67 |  |  |  |
| EIN2 | *A. thaliana* | 1,43* | 0,08 |  |  |  |
| HSP101 | *A. thaliana* | 1,92* | 0,35 |  |  |  |
| LUC | *A. thaliana* | 1,73* | 0,19 |  |  |  |
| GAPC | *N. benthamiana* | 1,48* | 0,17 |  |  |  |
| GFP | *N. benthamiana* | 2,02* | 0,2 |  |  |  |
| GUS | *N. benthamiana* | 1,69* | 0,05 |  |  |  |
|  |  |  |  |  |  |  |
| Legend: Induction of expression of several different proteins in different plants after treatments with corresponding cPEPs, | | | | | | |
| measured by Western blots. | |  |  |  |  |  |
| Asterisks indicate a significant difference between the test condition and the control according to the Wilcoxon test | | | | | | |
| ( n = 8; p < 0.05). | |  |  |  |  |  |

Supplementary Table 3

| Primer name | Primer sequence |
| --- | --- |
| LUC q5 | GCCGTTGTTGTTTTGGAGCA |
| LUC q3 | TGCGTCGAGTTTTCCGGTAA |
| AtActin q5 | GGTAACATTGTGCTCAGTGG |
| AtActin q3 | CTCGGCCTTGGAGATCCACA |
| Mtubi q5 | GCAGATAGACACGCTGGGA |
| Mtubi q3 | AACTCTTGGGCAGGCAATAA |
| MtNSP1 q5 | ATTCAACCAGTTCGGCATTC |
| MtNSP1 q3 | CTGCAAACCCTGCTTCTTTC |

Legend: List of primers used
