# Supplementary material for: Complementary peptides represent a credible alternative to agrochemicals by activating translation of targeted proteins
Source: Nat Commun. 2023 Jan 17;14:254. doi: 10.1038/s41467-023-35951-0 (PMC9845214; doi:10.1038/s41467-023-35951-0)
Supplement: Supplementary file 3 — Description of Additional Supplementary Files [file 41467_2023_35951_MOESM3_ESM.pdf]

Description of Additional Supplementary Files

File Name: Supplementary Data 1

Description: list of peptides used in this study.
